# An overview of rapid non-culture-based techniques in various clinical specimens for the laboratory diagnosis of *Talaromyces marneffei*

**DOI:** 10.3389/fcimb.2025.1591429

**Published:** 2025-05-23

**Authors:** Huamei Wei, Patcharin Thammasit, Artid Amsri, Kritsada Pruksaphon, Fenglian Deng, Joshua D. Nosanchuk, Sirida Youngchim

**Affiliations:** ^1^ Department of Microbiology, Faculty of Medicine, Chiang Mai University, Chiang Mai, Thailand; ^2^ Department of Pathology, The Affiliated Hospital of Youjiang Medical University for Nationalities, Baise, China; ^3^ Office of Research Administration, Chiang Mai University, Chiang Mai, Thailand; ^4^ Department of Medical Technology, School of Allied Health Sciences, Walailak University, Nakhon Si Thammarat, Thailand; ^5^ Department of Infectious Diseases, The Affiliated Hospital of Youjiang Medical University for Nationalities, Baise, China; ^6^ Department of Medicine (Division of Infectious Diseases), Department of Microbiology and Immunology, Albert Einstein College of Medicine, New York, NY, United States

**Keywords:** *Talaromyces marneffei*, talaromycosis, laboratory diagnosis, rapid diagnosis, nonculture-based techniques, clinical specimens

## Abstract

*Talaromyces marneffei* (*T. marneffei*) is a temperature-dependent biphasic deep opportunistic infectious fungus that primarily affects individuals with advanced HIV disease and other immunocompromised populations. Traditional diagnostic methods rely on fungal culture, but this process, although sensitive, is time-consuming and susceptible to contamination. Therefore, non-culture techniques serve as important complementary and alternative methods for diagnosing talaromycosis. They enable faster and more convenient pathogen identification, improving diagnostic efficiency and facilitating earlier initiation of treatment. Patients with talaromycosis can present with a wide range of clinical symptoms, and different clinical samples require different detection techniques. Blood samples are the most versatile, as laboratory technologists can utilize a wide range of diagnostic methods to obtain accurate results, particularly in the setting of a suspected disseminated infection. In contrast, urine diagnosis relies primarily on immunological methods that detect an antigen abundantly secreted during an infection. Moreover, for invasive samples like bronchoalveolar lavage fluid or cerebrospinal fluid, metagenomic next-generation sequencing is likely to be of significant importance for the early diagnosis due to its high sensitivity and specificity, though this approach is not yet standardized or widely available. For tissue samples, histopathology for light microscopy analysis is a well-established basic method, but it relies on experienced laboratory personnel, is time-consuming, and the histological appearance of other fungi can overlap with *T. marneffei*. Recent advances in rapid non-culture-based methods diagnostics underscore the growing importance of these tools in clinical settings, particularly for resource-limited areas where culture facilities are inadequate or unavailable. These methods improve diagnostic turnaround time and may lead to better clinical outcomes, especially for vulnerable patient populations. This review emphasizes the need for ongoing development and validation of non-culture diagnostics, with a focus on standardization, accessibility, and integration of rapid molecular and immunological tools to improve early detection and patient management in endemic regions.

## 1 Introduction

### 1.1 Originally and rapid non-culture diagnostics for *T. marneffei* infections: the paradigm shift

*Talaromyces marneffei* (*T. marneffei*), formerly known as *Penicillium marneffei*, is a thermally dimorphic fungus and an opportunistic pathogen endemic to Southeast Asia and southern China. First isolated from a bamboo rat in Vietnam in 1956 (Ajello et al., 1995; Pruksaphon et al., 2022a), it was recognized as a human fungal pathogen in 1973 (DiSalvo et al., 1973). *T. marneffei* primarily affects immunocompromised individuals, particularly those with HIV/AIDS, causing systemic and potentially life-threatening infections. *T. marneffei* infection in non-HIV patients is increasingly reported. Early detection, accurate diagnosis, and prompt antifungal therapy are essential for reducing morbidity and improving overall patient outcomes (Chan et al., 2019). The conventional laboratory diagnosis of *T. marneffei* relies on culture-based methods, which, while specific, are time-consuming and may delay treatment. In recent years, rapid non-culture-based techniques, including molecular assays, antigen detection, and serological tests, have emerged as valuable tools for early diagnosis. These methods enable faster and more sensitive detection across various clinical specimens, improving patient outcomes through timely antifungal therapy. This review provides an overview of these rapid diagnostic techniques, highlighting their clinical utility, advantages, and challenges in diagnosing *T. marneffei* infections.

### 1.2 Epidemiology in South and Southeast Asia

**Figure 1:**
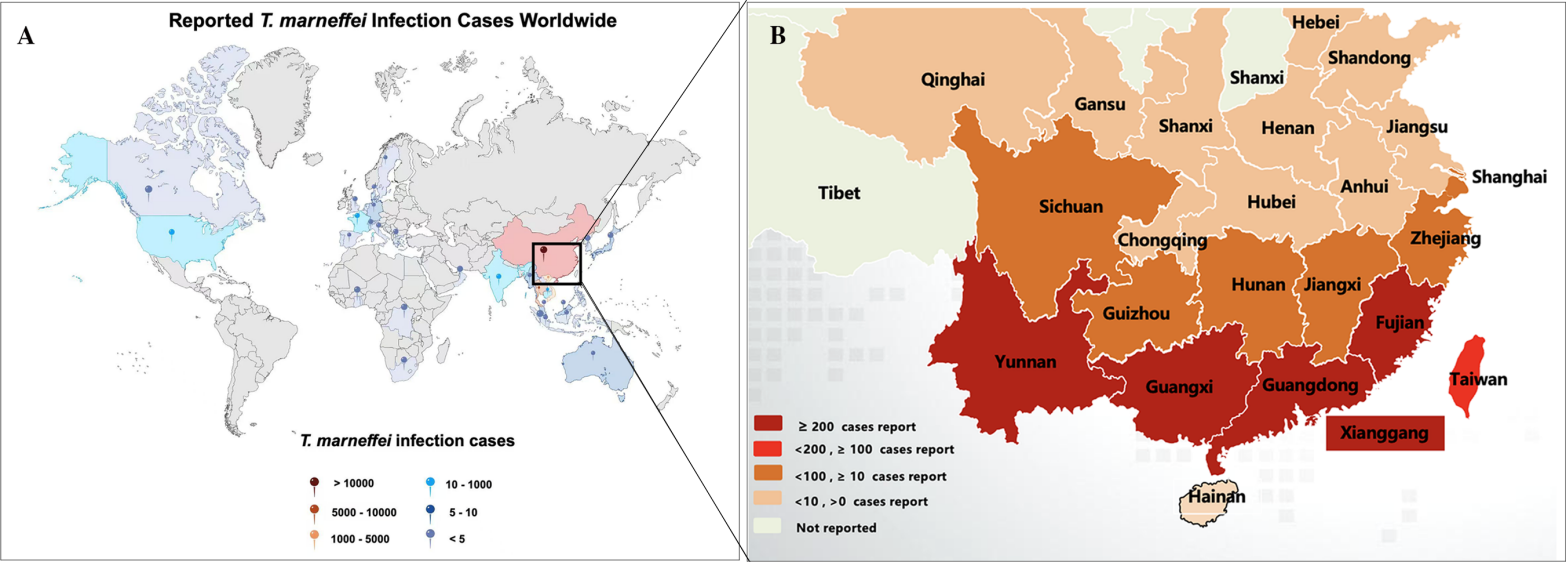
Reported talaromycosis cases worldwide and South-central China. **(A)** By mid-2022, talaromycosis was been reported in 34 countries. The picture was modified from Wang et al. (2023). **(B)** By mid-2024, *T. marneffei* infections were broadly distributed in south-eastern China. The graph was modified from Cao et al. (2019).

*T. marneffei* is endemic to the tropical regions of South and Southeast Asia. By mid-2022, over 288,000 cases of talaromycosis were recorded across 34 countries (**Figure 1A**) (Wang et al., 2023). Talaromycosis is most prevalent in China, Thailand, and Vietnam (Cao et al., 2019; Wang et al., 2023). In China, the regions with the highest incidence of *T. marneffei* are Guangxi and Guangdong (Hu et al., 2013). With the relocation of peoples in China, the disease has extended far beyond its original epidemic zone, leading to reports of talaromycosis in an additional 21 provinces and cities across China (**Figure 1B**) (Cao et al., 2019; Xu et al., 2023; Yang et al., 2024). HIV infection is closely associated with talaromycosis. For example, 1,079 patients with HIV-associated talaromycosis were evaluated in a single center in China, which revealed increases in both the number and prevalence of admissions in patients co-infected with talaromycosis and HIV from 125 (15.7%) in 2011 to 253 (18.8%) in 2017. The incidence of HIV-associated talaromycosis has increased in Guangdong, correlating with the high HIV burden in China (Ying et al., 2020). More recently, there has been a rise in *T. marneffei* infections among, for example, individuals with organ transplants or undergoing chemotherapy. He et al. (2021) reviewed 162 cases of *T. marneffei* infection in patients without HIV that were reported online from 2001 to 2019 and noted a significant upward trend in the infection rate of *T. marneffei* among both HIV and non-HIV populations. All these data highlight the importance of increasing awareness of *T. marneffei* infection, particularly in endemic regions.

### 1.3 Biology

**Figure 2:**
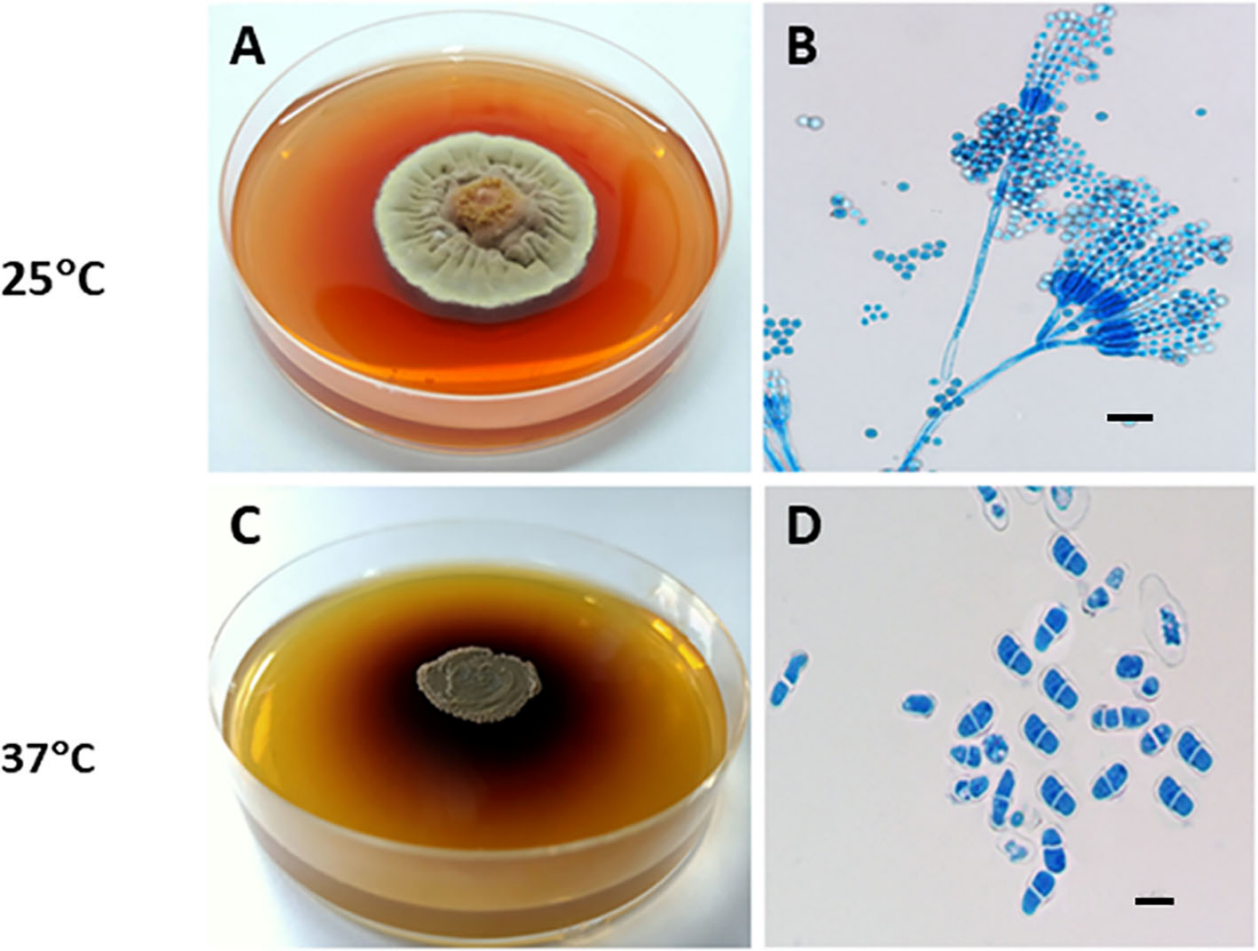
Thermal dimorphism of *T. marneffei* ATCC 200051. **(A)** At 25°C, *T. marneffei* grows on PDA as a mold, producing greenish-yellow to yellow conidia and secreting a distinctive diffusible red pigment; **(B)** Conidiophores have conidia chains that resemble those of other *Talaromyces* spp. (Magnification: 40x); **(C)** At 37°C, *T. marneffei* grows as a yeast with a dark-brown colony on BHI agar, producing a brown pigment; and **(D)** Yeast cells divide by fission. Bars, 5 μm (Magnification: 100x). This picture was chosen based on Dr. Youngchim’s description (Youngchim, 2022).

*T. marneffei* is a thermally dimorphic fungus, capable of growing as a saprophytic mold at room temperature and transitioning into a yeast form at mammalian body temperature. At room temperature (25–28°C), the fungus produces large amounts of conidia, which transform into fission yeast cells during human infection at 37°C. Mycelial colonies, like those of other *Talaromyces* spp., grow rapidly on Potato Dextrose Agar (PDA) at 25°C, forming flat, powdery white colonies. The colony generates diffusible red pigments in the agar and on its underside, which is a distinctive characteristic of *T. marneffei* (**Figure 2A**). The microscopic examination of the mycelia phase reveals typical morphology of *Penicillium* or *Talaromyces* species with characteristic dense brush-like, spore bearing structures (**Figure 2B**) (Vanittanakom et al., 2006).

At 37°C on Brain Heart Infusion (BHI) agar, *T. marneffei* undergoes a phase transition to yeast phase growth. Macroscopically, the yeast-like colonies present a cerebriform, convoluted, or smooth appearance. These glabrous, beige-colored colonies require 10 to 14 days to achieve full growth. *T. marneffei* yeast cultures produce a brown pigment (**Figure 2C**), not the red pigment generated by mycelial cultures. Microscopically, the yeast cells of *T. marneffei* are spherical to elliptical, separated by a single septum, measuring 2–3 to 2–6 μm (**Figure 2D**) (Youngchim, 2022).

**Figure 3:**
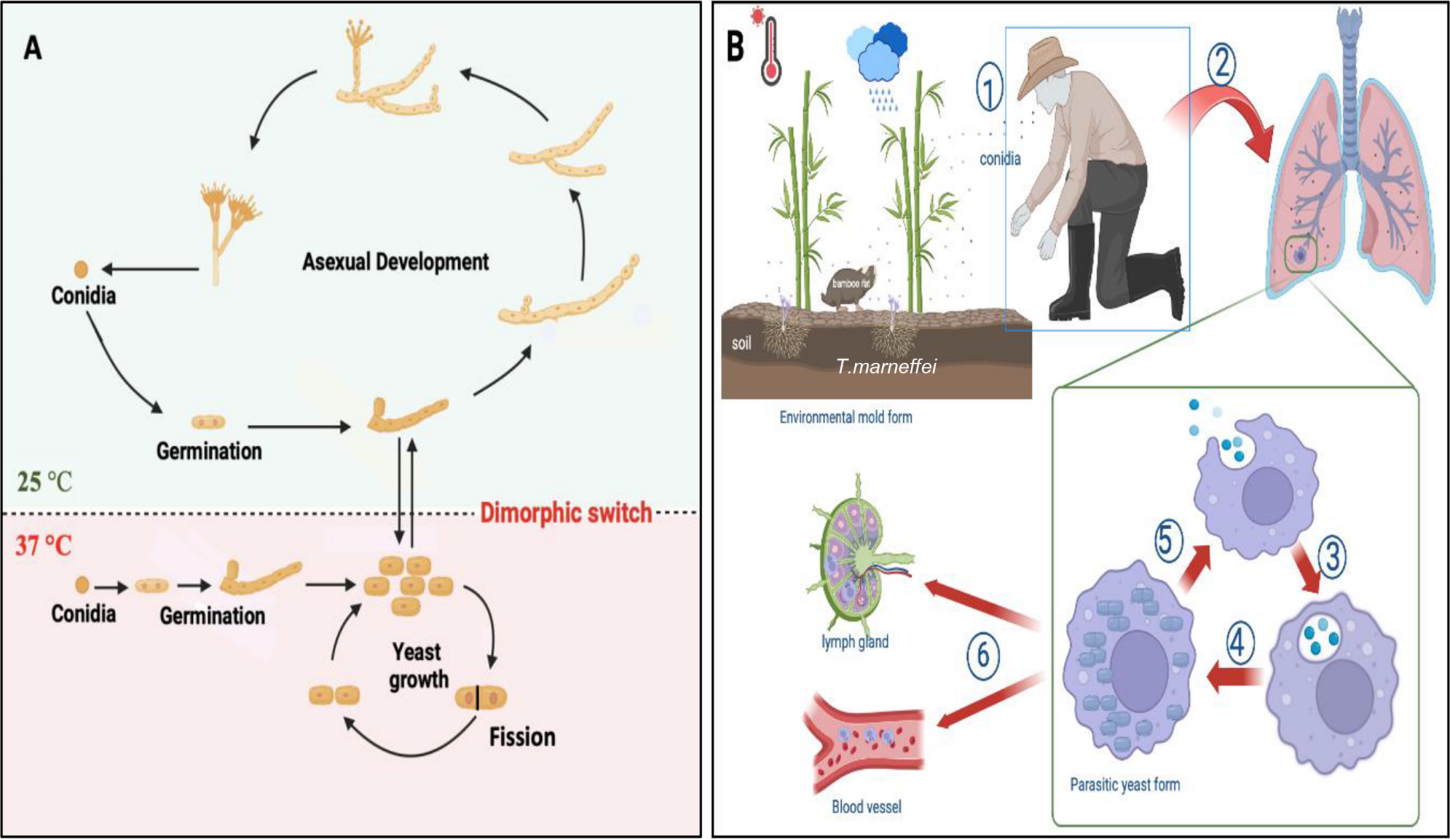
Morphogenesis and pathogenesis of *T. marneffei*. **(A)** Life cycle and morphologies of *T. marneffei*. At 25°C, conidia transitions into the filamentous phase of growth, characterized by an asexual cycle involving mycelial development and conidium production. At 37°C, conidia enter the yeast phase, which is characterized by yeast growth, and fission division. The primary dimorphic switch that connects the growth stages of yeast and mycelium is a response to temperature variation. The figure was modified from Andrianopoulos (2002); Wang et al. (2023) and visualized using BioRender.com. **(B)** The pathogenesis of *T. marneffei* infection: ① the host inhales conidia dispersed in the air; ② conidia enter the lung; ③ macrophages phagocytose conidia; ④ conidia transform into yeast cells in macrophages and continue to grow and reproduce by fission; ⑤ macrophages rupture, releasing the cells, which are then engulfed by other macrophages; ⑥ yeast cells can reside within macrophages, which then disseminate via the lymphatic system and bloodstream. The figure was adapted from Pruksaphon et al. (2022a) and visualized using BioRender.com.

In **Figure 3A**, *T. marneffei* conidia at 25°C are shown entering the filamentous phase to grow as multinucleate septate hyphae in a process of asexual development. Conidia can also directly germinate to form hyphae. Germination occurring at 37°C results in the conidium initially growing in a process of arthroconidiation, producing uninucleate yeast cells that undergo fission division. The dimorphic switching occurs not only in mammalian phagocytes but also in natural free-living amoebas, where temperature plays a crucial role in driving this phenomenon (Pruksaphon et al., 2022b).

The transmission route of *T. marneffei* to humans remains unclear. While inhalation of conidia is hypothesized to be the primary mode of infection, cutaneous inoculation via skin lesions may also occur (Wang et al., 2023). The conidia of *T. marneffei*, with a diameter of approximately 2 μm, are small enough to reach the alveoli of the lung, where they are subsequently engulfed by pulmonary histiocytes (Youngchim, 2022). Upon internalization, fungal conidia undergo direct conversion to fission yeast forms within these phagocytic cells (Pruksaphon et al., 2020a, 2023). Remarkably, *T. marneffei* yeast cells exhibit the capacity to survive and proliferate within this hostile intracellular environment, thus evading destruction by host immune cells. In immunocompromised hosts, once the fungus establishes itself intracellularly, yeast forms can disseminate throughout the body, consequently leading to systemic infection, as illustrated in **Figure 3B**.

Furthermore, macrophage polarization plays a critical role in the pathogenesis of *T. marneffei* (Shu et al., 2022). Macrophages differentiate into two distinct phenotypes: M1 (classically activation) and M2 (alternatively activation). The M1 phenotype, characterized by the expression of inducible nitric oxide synthase (iNOS), contributes to the formation and maintenance of granulomas, thereby limiting pathogen dissemination. Conversely, in immunodeficient individuals, such as those with human immunodeficiency virus (HIV) and CD4^+^ T cell counts below 50–100 cells/mm^3^, M2 macrophages may serve as a vehicle for the dissemination of *T. marneffei*, potentially leading to systemic infection. However, a rapid increase in CD4^+^ T cell levels following a period of depletion, as observed during antiretroviral therapy for HIV infection, can precipitate inflammatory conditions such as the immune reconstitution inflammatory syndrome (IRIS). This paradoxical response underscores the complex interplay between the host immune system and *T. marneffei* infection (Wang et al., 2023; Pruksaphon et al., 2024a).

### 1.4 Clinical manifestation

*T. marneffei* infections most frequently occur in patients with advanced HIV infection where they manifest as severe and often fatal systemic infections (Vanittanakom et al., 2006). *T. marneffei* mainly affects organs and tissues related to the monocyte–macrophage reticuloendothelial system. Focal infections are generally confined to the site of invasion, with clinical manifestations primarily reflecting the symptoms of the underlying disease (Peng et al., 2022). In contrast, disseminated infections involve multiple tissues and organs. The clinical symptoms of disseminated *T. marneffei* infection are often nonspecific, which can lead to misdiagnoses as tuberculosis or other fungal infections. However, patients typically present with fever, cough, lymph node enlargement, weight loss, anemia, skin lesions, and various organ-specific symptoms (Castro-Lainez et al., 2018). Kawila et al. reported that cutaneous manifestations are different in HIV co-infection compared to talaromycosis in the absence of HIV disease as 95.7% HIV patients developed umbilicated skin lesions whereas 35.7% of those without HIV infection had sweet’s syndrome. Hence, the occurrence and appearance of skin manifestations are related to the immune status of the patient (Kawila et al., 2013).

### 1.5 Laboratory diagnosis

Traditionally, the diagnostic gold standard for diagnosing talaromycosis has relied on culturing the organism from clinical specimens and observing its morphological features under a microscope (Castro-Lainez et al., 2018). However, this approach is time-consuming (several days to >2 weeks), which is less than ideal given the rapid progression of the infection in vulnerable patients. Therefore, there is a critical need for non-culture-based, accurate, rapid, and cost-effective diagnostic methods to improve patient outcomes.

Non-culture-based diagnostic techniques have led to the development of various diagnostic techniques that provide faster *T. marneffei* detection. For example: polymerase chain reaction (PCR), enzyme-linked immunosorbent assays (ELISA) and next-generation sequencing (NGS) methods have shown high sensitivity and specificity, and these approaches can significantly reduce the time required to diagnose *T. marneffei* infection (Ning et al., 2018). PCR techniques, such as real-time PCR (qPCR), nested PCR, and *in situ* hybridization PCR, can provide results within hours as opposed to days or weeks required for culture-based approaches. Other immunological and various biochemical criterion tests have also been employed to detect specific antigens or antibodies associated with *T. marneffei*.

Despite significant advancements in diagnostic technologies, the selection of an appropriate diagnostic method for *T. marneffei* often relies on reliability of results, difficulty and ease of operation, available resources, the clinical setting, and specific characteristics of the patient’s condition (Ning et al., 2018; Pruksaphon et al., 2020b). Crucially, there is no universal consensus on the optimal rapid diagnostic technique due to variations in sensitivity and specificity among different methods, particularly when different types of clinical specimens are considered. Each specimen type, ranging from blood, serum, plasma and urine to more invasive samples like CSF and tissue biopsies, may require distinct processing and diagnostic approaches to accurately detect *T. marneffei*. Thus, the diagnosis of disseminated *T. marneffei* infection in patients remains challenging in clinical practice. This review aims to summarize non-culture based diagnostic techniques for rapid detection and to identify the most effective methods across various clinical specimen types of *T. marneffei* infection.

## 2 Non-culture-based techniques for *T. marneffei* detection

Non-culture-based techniques have gradually become important complementary and alternative means for the diagnosis of *T. marneffei.* These techniques included molecular biology, serological testing, imaging and pathology methods that enable faster and more accurate identification of pathogens and improve diagnostic efficiency.

### 2.1 Molecular biology methods

Molecular biology methods are essential for the non-culture diagnosis of *T. marneffei*. Molecular methods mainly utilize PCR and its derivative technologies, such as nested PCR, quantitative PCR, loop-mediated isothermal amplification (LAMP) and metagenomic next-generation sequencing (mNGS). These methods enable direct identification of pathogens by amplifying and detecting specific DNA or RNA sequences of *T. marneffei.* PCR and its derived techniques have the advantages of high sensitivity and specificity, allowing rapid identification of infection at an early stage. mNGS, on the other hand, can detect all microbial sequences in a sample without bias, providing comprehensive information for the diagnosis of complex infections (Zheng et al., 2021).

#### 2.1.1 PCR

The PCR is a process conceived by Kary Mullis in 1983, and it is a widely recognized and highly effective technique for the detection of many infectious diseases in clinical samples, including fungal infections like *T. marneffei* (Zhu et al., 2018). At present, specific oligonucleotide primers designed from the 5.8S rRNA and 18S rRNA genes of *T. marneffei* are commonly used for sequence determination in the internal transcribed spacer (ITS), which contains both conserved and specific regions (Lei et al., 2018). By sequencing ITS regions, β-tubulin genes, etc., each isolation can be specific (Cao et al., 2019). Although conventional PCR has high sensitivity and good specificity, it remains labor-intensive and there are other limitations, such as post-PCR steps. Additionally, obtaining the genomic DNA required for detection necessitates isolation and culture of the pathogen, a process that typically takes ~5 days.

#### 2.1.2 Quantitative PCR

Quantitative PCR (qPCR) provides information on the presence as well as the amount of a pathogen. The sensitivity of fluorescence qPCR with clinical *T. marneffei* patients was 86.11%, which was more sensitive than a blood culture method, and the approach could be used as a simple method for DNA quantification of *T. marneffei* (Li et al., 2020). Moreover, whole blood and plasma samples were evaluated by qPCR without contamination, however the method requires significant time and technical effort by laboratory staff (Hien et al., 2016; Lu et al., 2016). The method can also be used to detect *T. marneffei* on patient’s skin from pathology section and lymph node biopsy (Zheng et al., 2018). Although the time to diagnosis using skin pathological sections from *T. marneffei* infected patients was shorter than culture-based technique, the pathology processing was time-consuming and complex. Thus, although qPCR assay offers a more sensitive tool to detect *T. marneffei* compared to conventional PCR, there are some limitations regarding the improvement of its speed and high specificity for qPCR.

#### 2.1.3 Nested-PCR

Gel-based nested PCR is a method for efficient DNA amplification. In a comparison study of nested PCR and real-time PCR to detect *T. marneffei* DNA in whole blood samples from 26 HIV-infected patients, the sensitivity and specificity detection rates for the nested PCR assay was 95% and 75%, while the real-time PCR assay was 89% and 63%, respectively, suggesting that nested PCR was a more effective approach for identifying *T. marneffei* DNA in whole blood (Lu et al., 2016). For paraffin-embedded tissue samples, the limit of detection (LOD) for the single PCR approach using specific primers Pm1 and Pm2 was 14 pg/μl, while the LOD for nested PCR with primer pairs RRF1 and RRH1, as well as Pm1 and Pm2, was 14 fg/μL (Zeng et al., 2009). Using bronchoalveolar lavage fluid (BALF), Liu et al. (2016) confirmed that nested PCR assay had a high predictive value in reflecting pulmonary infection with a positive rate of 83.3%. In the clinic, lung biopsies are not routinely performed because this invasive procedure may worsen the patient’s respiratory function whereas BALF can be obtained through less invasive procedures, which are generally more favorable for patients. Although nested PCR has advantages in improving detection sensitivity and specificity in *T. marneffei*, it is relatively complex to operate, requiring at least two rounds of PCR reactions using two sets of primers (external and internal primers), which increases the complexity and time of the test.

#### 2.1.4 Loop-mediated isothermal amplification

LAMP is a simple, rapid, and specific method for nucleic acid amplification. A LAMP assay has been piloted with pure cultures as well as in paraffin wax-embedded tissues, and the method can be carried out within 1 hour (Sun et al., 2010). However, a LAMP-based diagnostic method has not progressed to date for *T. marneffei*, possibly due to challenges such as complex primer design, intricate product structures, and difficulties in quantification.

#### 2.1.5 Metagenomic next-generation sequencing

In recent years, advances in metagenomic next-generation sequencing (mNGS) have significantly enhanced the ability to detect pathogenic microorganisms (Wei et al., 2022; Zhang et al., 2024). mNGS, a high-throughput sequencing technology, enables direct extraction and analysis of DNA/RNA from clinical specimens. This methodology facilitates comprehensive pathogen detection through comparative analysis with specialized databases and subsequent bioinformatics processing, allowing for simultaneous, unbiased identification of *T. marneffei* without the need for pre-defined parameters or cultivation.

mNGS can simultaneously screen for multiple pathogens without the need for high-quality DNA extraction or specific primers. This provides a comprehensive view of the infectious landscape, which is particularly beneficial in diagnosing complex cases with potential co-infections. Unlike PCR, which requires sufficient pathogen DNA and may miss low-abundance pathogens, mNGS can identify *T. marneffei* in samples with low pathogen loads or degraded DNA, and it avoids the challenges associated with centrifugation and sample processing. In a Chinese cohort, mNGS, has proven highly effective in detecting *T. marneffei* in patients with inborn errors of immunity (IEI) using blood samples, achieving a sensitivity of 100% and a specificity of 98.7%, with results available in about 26 hours (Liu et al., 2022b). An unusual case of infective endocarditis caused by *T. marneffei* in an elderly patient also benefited from the rapid diagnosis of mNGS using blood samples in the early diagnosis and management of uncertain microbial infections (Ji et al., 2024). In non-HIV patients, mNGS continues to demonstrate rapid and unbiased diagnostic potential, particularly after negative results from blood cultures (Yang et al., 2022). Nevertheless, the high cost restricts the application of mNGS in large patient cohorts.

Overall, the detection of *T. marneffei* using PCR or PCR-based molecular techniques demonstrates high sensitivity and specificity. However, obtaining high-quality DNA is a prerequisite for the successful application of these methods. Due to the typically low quantity of *T. marneffei* yeast cells in many tissues and fluids, obtaining sufficient high-quality DNA remains a major challenge. Additionally, yeast cells may be lost during serum processing due to centrifugation. Other challenges include the need for highly trained personnel, the complexity and cost of primers, and the varying processing requirements for different tissue and fluid samples.

### 2.2 Histopathology

Diagnosis of talaromycosis by histopathology can be achieved using materials from skin to deep tissues. Histopathological examinations are available within 1 day to 2 weeks depending on the laboratory, tissue type, and stains, offering a potentially faster alternative to the more time-consuming culture-based methods. Histopathology enables direct visualization of pathological changes and pathogen-specific features in tissue samples, facilitating timely intervention based on accurate diagnosis. Grocott’s methenamine silver stain (GMS), Periodic Acid–Schiff (PAS) and Hematoxylin and Eosin (H&E) staining of histopathological sections can reveal the characteristic yeast cells of *T. marneffei*, typically round or oval within macrophages (Yousukh et al., 2004). However, a major challenge of histopathology using routine stains is misidentification of the fungus, for example as histoplasmosis (Widaty et al., 2020). Further, the preparation time for the tissues section can be long and relatively complicated, and staining techniques can also be difficult to properly perform. Moreover, accurate microscopic analysis demands skilled laboratory professionals and often requires verification by at least two independent analysts to confirm the results.

Additionally, Wright-Giemsa staining (WGS) applied to specimens from fine-needle aspirations of lymph nodes or bone marrow, smears of skin, or lymph node biopsies can highlight basophilic, spherical, oval, and elliptical yeast cells with a central septation, a feature distinctively associated with *T. marneffei* and different from the budding patterns seen in other clinically significant yeasts (Qin et al., 2015). *In situ* hybridization (ISH) is the application of a nucleic acid probe with a known base order and a marker to specifically combine nucleic acids to be detected in tissues and cells according to the principle of base-pairing to form a hybrid. The detection system corresponds to the marker to form a colored hybridization signal in the nucleic acids detected *in situ* by histochemistry or immunohistochemistry, and to locate them intracellularly under a microscope or an electron microscope (Levsky and Singer, 2003). This method can be used to observe the strong staining of *T. marneffei* cell wall, clear contour, and easy to distinguish from surrounding tissues in tissue sections. The approach can effectively detect *T. marneffei* in tissue sections, overcoming the shortcomings of conventional pathological staining and microscopic examination of pathological sections, which require auxiliary diagnostic tools to identify *T. marneffei* due to its atypical shape and size variation.

## 3 Non-DNA-based rapid techniques for *T. marneffei* detection

Besides molecular tests detection methods and histopathology, immunological testing is commonly used for the rapid diagnosis infectious diseases using clinical samples, especially blood samples. These tests utilize the specific interactions between antibodies and antigens to quickly identify and measure specific pathogens or their byproducts in human body fluids, enabling rapid disease diagnosis. Common immunological testing methods, including ELISA, ELISA-based detection technology, Latex agglutination test (LA), and Immunochromatographic test (ICT), have the advantages of being fast, sensitive, simple and easy to standardize, leading to their frequent and wide use for infection diagnosis.

### 3.1 ELISA

ELISA is an immunoassay technique characterized by high sensitivity, easy handling, precise and intuitive results, and a wide range of applications. ELISA can detect both antibodies and antigens, perform qualitative and quantitative analyses, and localize and analyze specific antigens. Using the rabbit polyclonal antibodies (PAbs) to heat-killed whole-fragment arthroconidia of *T. marneffei*, Desakorn and colleagues developed a combination dot blot ELISA and ELISA assay to detect *T. marneffei* urinary antigens with sensitivities of 94.6% and 97.3%, respectively (Desakorn et al., 2002). A potentially immunogenic protein, designated Mp1p, was identified in the mycelium and yeast phase cell walls of *T. marneffei* and determined to be encoded by the *MP1* gene. Purified recombinant Mp1p was used in an ELISA to detect antibodies in patients with Acquired Immunodeficiency Syndrome (AIDS) with culture-confirmed *T. marneffei* infection with 100% specificity and 82% sensitivity (Cao et al., 1999). The PAbs to Mp1p mannoprotein were also incorporated into a sandwich ELISA to detect the Mp1p antigen in patient sera with a sensitivity of 65%. However, a combination of Mp1p antigen and anti-Mp1p antibody detection improved the diagnostic sensitivity to 88%. The researchers suggested that both tests be performed for patients in whom talaromycosis is suspected. The antibody test may be more sensitive for patients who are immunocompetent or who have better humoral immune systems, while the antigen test would be more useful for patients who have more compromised immune systems (Cao et al., 1999). However, cross reactivity was found with antigens from non-pathogenic *Penicillium pinophilum* and from individuals with *Cryptococcus neoformans* or *Candida* spp. as well as from healthy controls in *T. marneffei* endemic areas (Chaiyaroj et al., 2003). Although ELISA has the characteristics of high sensitivity, specificity, quantitative detection, wide application range and good repeatability to detect *T. marneffei*, false positive results and cross-reactions remain major obstacles that must be addressed prior to the routine clinical application of these methods.

### 3.2 Latex agglutination test

LA is a rapid indirect agglutination test using latex particles as a carrier for an antibody to capture antigen from a clinical specimen. This method has several important advantages over the tests previously described, including no need for special instruments, visual judgment, easy operation, and no requirement for advanced training. The detection time is short, usually within 2 minutes. The cost of testing a single serum sample is much lower than other serological and microbiological based methods. LAs are also suitable for field detection. Given these positive characteristics, LAs are widely used in clinical testing. The LA using the same rabbit PAbs to heat-killed whole-fragment arthroconidia of *T. marneffei* described was shown to detect *T. marneffei* in urine. Compared with a previously validated ELISA, the LA produced the highest sensitivity (100%) and specificity (99.3%), demonstrating that the assay was a simple, robust, and rapid method that could effectively enhance the diagnostic toolkit for talaromycosis (Desakorn et al., 2002).

### 3.3 Immunochromatographic tests

ICT is a commonly used biotechnological method to detect and analyze interactions between specific antigens and antibodies using highly specific combinations of antigen and antibody to achieve the detection and quantitative analysis of the target. ICT is now available for the immunodiagnosis of various mycoses, including cryptococcosis (Sathongdejwisit et al., 2021; Pruksaphon et al., 2024b), aspergillosis (Thornton, 2008; Jenks et al., 2020) and talaromycosis (Pruksaphon et al., 2018, 2021). Pruksaphon et al. established a prototype ICT for the detection of *T. marneffei* cytoplasmic yeast antigens in urine samples. The highly *T. marneffei*-specific monoclonal antibody 4D1 (MAb 4D1) conjugated with gold colloid at pH 6.5 was used as signal generator. Subjecting the assembled test strip to urine samples containing *T. marneffei* antigen produced a visible result within 20 minutes. The limit of detection (LOD) for ICT was 3.125 μg/mL of TM CYA. The test exhibited sensitivity, specificity, and accuracy of 87.87%, 100% and 95.50%, respectively. ICT is a rapid, user-friendly test which holds great promise for the serodiagnosis of *T. marneffei* infection.

### 3.4 Galactomannan assay

GM is a heteropolysaccharide composed of a non-immunogenic mannan core and immunodominant galactofuranosyl side chains, primarily found in the cell walls of genus *Aspergillus* and genus *Talaromyces* (Huang et al., 2007). A commercial serum GM assay (Platelia *Aspergillus* Enzyme Immunoassay Kit) has been shown to be an effective tool for the early serodiagnosis of *T. marneffei* infection in a people living with HIV/AIDS (PLWHA) (Zheng et al., 2015). Moreover, GM levels can be used to assess clinical response and fungal clearance during antifungal treatment. However, the cross-reaction between the *Aspergillus* GM assay and *Talaromyces* GM limits the clinical application (Pruksaphon et al., 2020b). Since aspergillosis is rare among AIDS patients compared to talaromycosis, the GM assay remains a valuable tool for diagnosing *T. marneffei* disease.

### 3.5 1,3-β-D-glucan assay

The 1,3-β-D-glucan (BDG) is a key structural component of the cell wall in most pathogenic fungi, with the exception of Mucorales (Zygomycetes) fungi and *Cryptococcus* spp. As a result, unlike the GM assay, which is specific to certain fungi, BDG can typically be detected in both invasive aspergillosis and candidiasis. This is particularly relevant for patients with hematologic malignancies and neonatal disorders (Fontana et al., 2012). Currently, 4 commercial assay kits are available for BDG detection: Fungitell, Fungitec-G, Wako, and Maruha (Lamoth et al., 2012; Ferreras-Antolin et al., 2022). The BDG test is considered positive when the concentration is ≥ 0.1 ng/ml (Liu et al., 2022; Gourav et al., 2024). In cases of talaromycosis, elevated serum BDG levels were observed in 82% of patients in a study conducted in Japan (Yoshimura et al., 2016). Notably, these patients had a history of travel to endemic areas. Given that BDG elevation was present in the majority of cases, this assay may serve as a useful diagnostic tool for identifying *T. marneffei* infections in individuals residing in non-endemic areas who have recently traveled to endemic zones. However, it is important to note that the study included only 11 participants, which may limit the statistical power and generalizability of its findings (Zaongo et al., 2023).

## 4 Specimen types

### 4.1 Bronchoalveolar lavage fluid

BALF is a direct pulmonary specimen obtained via bronchoscopy that provides valuable insights into localized lung infections, including those caused by *T. marneffei.* Since the pathogen primarily affects the lungs, BALF is particularly effective for its detection. Due to its direct sampling from the lower respiratory tract, BALF offers higher sensitivity and specificity for identifying pulmonary pathogens, compared to blood or urine samples. Therefore, BALF serves as a crucial diagnostic tool for pulmonary infections, particularly when less invasive tests fail to provide definitive results (Hogea et al., 2020).

BALF specimens for detection of *T. marneffei* have been study by mNGS as this technique has demonstrated significant advantages in identifying pathogens in cases of unclear infections (Yang et al., 2022; Ji et al., 2024). In a study involving six patients, mNGS diagnosed *T. marneffei* in two cases, where multiple traditional cultures failed to detect the pathogen. Specifically, mNGS identified *T. marneffei* reads in one BALF sample, and another in an intestinal lesion, with the pathogen also being detected in their blood samples (Peng et al., 2022). High-throughput sequencing of a BALF sample was used to diagnose *T. marneffei* infection in a pediatric patient, which was subsequently confirmed by BALF culture (Lin et al., 2022). Although PCR-based methods, ELISA, and GM measurements have been applied to testing BALF in the setting of other mycoses, these approaches have not been rigorously studied for the detection of *T. marneffei* in BALF. Interestingly, the application of liquid-based cytopathology (LCT) with GMS staining on BALF has offered a superior diagnostic approach for identification of *T. marneffei*, which indicates that GMS staining in LCT could serve as a valuable rapid method for enhancing the diagnosis (Hu et al., 2020), while the sensitivity and specificity metrics should also be evaluated in further studies.

### 4.2 Blood samples

Blood samples are among the most common and readily available specimens in clinical settings and are currently the most widely used specimens for a broad array of rapid diagnostics. The rapid diagnosis of *T. marneffei* from blood samples is crucial for timely and effective treatment.

**Whole blood** – Whole blood plays a crucial role in the interaction with *T. marneffei*, a facultative intracellular dimorphic fungus capable of surviving and replicating within mononuclear cells (Pruksaphon et al., 2022a). Mononuclear cell and macrophages in whole blood create a suitable environment for *T. marneffei* to complete its dimorphic transition and persist, emphasizing the role of blood as a medium for fungal propagation (Prakit et al., 2016). **Table 1** presents a comparative analysis of various detection techniques for *T. marneffei* in whole blood samples. Among these, metagenomic next-generation sequencing (mNGS) demonstrated superior performance, achieving both 100% sensitivity and specificity (Mao et al., 2022). In the comparison with PCR-based methods, nested PCR exhibited the highest sensitivity (94.73%), followed by quantitative PCR (qPCR) (89.47%), and TaqMan qPCR (60.00%) (Pornprasert et al., 2009; Lu et al., 2016). The enhanced sensitivity of nested PCR can be attributed to its two-stage amplification process. Conversely, TaqMan qPCR demonstrated superior specificity (100%) compared to nested PCR (75.00%) and qPCR (63.50%), likely due to its utilization of *T. marneffei*-specific primers and probes. The relatively low specificity observed in qPCR and nested PCR may be attributed to the complex composition of whole blood, which can potentially interfere with amplification processes. Despite the high sensitivity and specificity of mNGS, its widespread implementation is currently limited by its substantial cost. It is noteworthy that the intricate composition of whole blood, particularly its high protein content, significantly impacts the sensitivity and specificity of PCR-based assays (Sidstedt et al., 2018). This complex matrix effect underscores the challenges in developing reliable molecular diagnostic tools for *T. marneffei* detection in whole blood specimens. Consequently, while mNGS shows promise, the high cost limit its use, while the current PCR-based methods for *T. marneffei* detection in whole blood samples demonstrate suboptimal performance in terms of sensitivity and specificity, highlighting the need for further refinement and optimization of these techniques (Al-Soud and Rådström, 2001).

**Table 1:** Sensitivity and specificity of different detection techniques in whole blood samples.

Sample Type	Patients	Techniques	Targets	Sensitivity (%)	Specificity (%)	Time	Ref
Whole blood	26 culture-proven TM patients (19 HIV+, 3 HIV-)	qPCR	ITS2	89.47 (17/19)	63.50 (5/8)	N/A	(Lu et al., 2016)
		Nested-PCR	ITS4/5 and ITS2	94.73 (18/19)	75.00 (6/8)	N/A	
	13 culture-proven TM patients (HIV +)	mNGS	Genomic DNA	100.00 (13/13)	100.00 (22/22)	1–2 days	(Mao et al., 2022)
	20 culture-proven TM patients	TaqMan qPCR	5.8S rRNA gene	60.00 (12/20)	100.00 (20/20)	<1 day	(Pornprasert et al., 2009)

N/A, Not available.

**Serum** - Serum is the most widely used specimen type for testing *T. marneffei* in patients with AIDS because it is readily available compared to whole blood and plasma. **Table 2** shows the performance of different tests for diagnosing talaromycosis using serum. Comparing qPCR (86.11%, 100%) and Nested-PCR (68.57%, 100%), the sensitivity of qPCR was higher than Nested-PCR. Notably, the sensitivity and specificity of qPCR for detecting *T. marneffei* in serum is higher than in whole blood, suggesting that serum is the preferred blood fluid for this approach. In contrast, the results with Nested-PCR were better in whole blood better than serum. In terms of immunological studies, Mp1p is a well-known mannoprotein of *T. marneffei*, known for its immunogenic properties as both a surface and secretory form (Woo et al., 2016; Ly et al., 2020; Thu et al., 2021; Chen et al., 2022; Gong et al., 2023; Li et al., 2024). An ELISA using PAb-MAb against Mp1p ELISA plus an anti-Mp1p IgG antibody ELISA resulted in a sensitivity and specificity of 100% and 98%, respectively. Mp1p shows high sensitivity and specificity, however, Mp1p is not a specific protein of only *T. marneffei* and cross-reactivity occurs (Wang et al., 2011). Notably, the sensitivity and specificity of monoclonal antibody 4D1 (MAb 4D1) detection by inhibition ELISA are 100% (Prakit et al., 2016).

**Table 2:** Sensitivity and specificity of different detection techniques in serum samples.

Sample Type	Patients	Techniques	Targets	Sensitivity (%)	Specificity (%)	Time	Ref
Serum	36 culture-proven TM patients (14 HIV+, 22 HIV-)	qPCR	ITS1-5.8S- ITS2 rDNA	86.11 (31/36)	100.00 (75/75)	N/A	(Li et al., 2020)
		GM assay	Galactomannan	80.56 (29/36)	45.33 (34/75)	N/A	
	26 culture-proven TM patients (24 HIV+, 2 HIV-)	ELISA	Mp1p antigen	88.46 (23/26)	100.00 (85/85)	N/A	(Cao et al., 1999)
	24 culture-proven TM patients (HIV+)	GM assay	Galactomannan	95.83 (23/24)	90.91 (30/33)	3 hours	(Zheng et al., 2015)
	93 culture-proven TM patients (HIV+)	GM assay	Galactomannan	92.63 (176/190)	64.52 (60/93)	N/A	(Chen et al., 2022)
		Enzyme immunoassay	Mp1p antigen	72.04 (67/93)	96.84 (184/190)		
	20 culture-proven TM patients	MAb-MAb ELISA	Mp1p antigen	55.00 (11/20)	99.62 (523/525)	N/A	(Wang et al., 2011)
		PAbs-MAb ELISA	Mp1p antigen	75.00 (15/20)	99.43 (522/525)		
		Anti-Mp1p IgG ELISA	Mp1p specific antibodies	30.00 (6/20)	98.48 (517/525)		
		MAb-MAb ELISA + Anti-Mp1p IgG ELISA	Mp1p antigen and Mp1p specific antibodies	85.00 (17/20)	98.10 (515/525)		
		PAbs-MAb ELISA + Anti-Mp1p IgG ELISA	Mp1p antigen and Mp1p specific antibodies	100.00 (20/20)	97.90 (514/525)		
	95 culture-proven TM patients (HIV+)	Double-antibody sandwich ELISA	Mp1p antigen	71.58 (68/95)	97.25 (248/255)	N/A	(Gong et al., 2023)
	60 culture-proven TM patients (HIV+)	GM assay	Galactomannan	83.33 (50/60)	91.89 (136/148)	1–2 days	(Mao et al., 2022)
	17 culture-proven TM patients (HIV+)	ELISA	Mp1p antibody	82.35 (14/17)	100.00 (90/90)	N/A	(Cao et al., 1998)
	45 culture-proven TM patients (HIV+)	Inhibition ELISA using MAb 4D1	Cytoplasmic yeast antigen	100.00 (45/45)	100.00 (45/45)	N/A	(Prakit et al., 2016)
	3 culture-proven TM patients (HIV+)	qPCR	ITS2	100.00 (3/3)	100.00 (3/3)	N/A	(Lu et al., 2016)
		Nested-PCR	ITS4/5, ITS2	0.00 (0/3)	0.00 (0/3)		
	15 by microscopy and cultures-proven TM patients (HIV+)	GM	Galactomannan	66.67 (10/15)	90.91 (30/33)	<1 days	(Huang et al., 2007)
	17 culture-proven TM patients (HIV+)	Immunodiffusion test	*T. marneffei* antigens	58.82 (10/17)	100.00 (21/21)	N/A	(Kaufman et al., 1996)
		Latex agglutination test	*T. marneffei* antigens	76.47 (13/17)	100.00 (21/21)		
	35 culture-proven TM patients (HIV+)	Nested PCR	RRF1, RRH1; Pm1, Pm2	68.57 (24/35)	100.00 (365/365)	N/A	(Pongpom et al., 2009)

**Plasma** - **Table 3** presents the performance of different tests using plasma. Interestingly, Mp1p antigen-ELISA demonstrates superior sensitivity (86.29%) compared to TaqMan real-time PCR (70.37%) for *T. marneffei* detection in plasma samples. The Mp1p antigen-ELISA methodology offers advantages in terms of simplicity, convenience, and cost-effectiveness relative to TaqMan real-time PCR. However, it is noteworthy that plasma is infrequently employed as a specimen type in research settings due to the presence of anticoagulants such as ethylenediaminetetraacetic acid (EDTA) or heparin. EDTA presents significant challenges in both ELISA and PCR-based assays. In ELISA, EDTA can interfere with the alkaline phosphatase reaction, potentially compromising the assay’s performance. In molecular diagnostic techniques, EDTA may form complexes with certain metal ions, thereby impeding DNA extraction and amplification processes (Lopata et al., 2019). Heparin, another commonly used anticoagulant, also introduces complications in both TaqMan PCR and ELISA methodologies. In TaqMan PCR, heparin can bind to DNA or RNA targets, thereby inhibiting the specific binding of the TaqMan probe and potentially leading to false-negative results. Similarly, in ELISA, heparin can interfere with the assay by binding to antigens or antibodies, thus affecting their capacity to bind specifically to their respective targets (Willems et al., 1993; Satsangi et al., 1994). These anticoagulant-induced interferences underscore the complexities associated with utilizing plasma samples for *T. marneffei* detection and highlight the need for careful consideration of specimen types and potential confounding factors in diagnostic assay development and implementation.

**Table 3:** Sensitivity and specificity of different detection techniques in plasma samples.

Sample Type	Patients	Techniques	Targets	Sensitivity (%)	Specificity (%)	Time	Ref
Plasma	50 culture-proven TM patients (HIV+)	Real-time PCR	*MP1* gene	70.37 (19/27)	100.00 (20/20)	5–6 hours	(Mao et al., 2022)
	372 culture-proven TM patients (HIV+)	Enzyme immunoassay	Mp1p antigen	86.29 (321/372)	98.07 (507/517)	6 hours	(Thu et al., 2021)

**Selection of blood fraction** - Currently, there is no consensus on the most effective blood fraction for isolating fungal DNA for PCR analysis. Studies have indicated that plasma PCR exhibits greater sensitivity compared to PCR performed on whole blood or serum. Conversely, serum PCR has demonstrated comparable or even superior performance relative to whole blood PCR. In one study comparing nested PCR and real-time PCR assays to detect *T. marneffei* DNA in whole blood samples from 23 HIV-infected patients, the positive detection rate for the nested PCR assay was 67%, while the Real-Time PCR assay was 77%, suggesting a combination of nested and Real-Time PCR assays is a potential approach for identifying *T. marneffei* DNA in whole blood samples from HIV-infected patients (Lu et al., 2016). The *MP1* gene has also been targeted for the development of Real-Time PCR for *T. marneffei* using human plasma, resulting in a specificity and sensitivity of 70.37 and 100%, respectively (Hien et al., 2016). Another study by Li et al. (2020) demonstrated that the utility of qPCR targeting ITS1-5.8S-ITS2 combined with a GM assays offer a highly sensitive and reliable approach for diagnosing *T. marneffei* infection using serum samples for DNA quantification in endemic areas. These studies provide preliminary references for PCR testing of *T. marneffei* DNA across different blood components and suggest that combining multiple PCR methods can enhance sensitivity and accuracy.

### 4.3 Urine

Urine samples are simple to acquire and urine is usually abundant. However, even though *T. marneffei* predominantly causes systemic infections, the concentration of its DNA in urine may be too low for detection (Chen et al., 2018). Additionally, urine contains various inhibitors that can interfere with the PCR process, such as urea, creatinine, and other substances that can degrade DNA or inhibit enzyme activity necessary for the PCR reaction (Hosbrough, 2020). These factors collectively contribute to making urine a less favorable sample for PCR-based diagnostics of *T. marneffei* infections. To date, there have been no reports on the use of PCR-based methods for the rapid diagnosis of *T. marneffei* in urine samples. Instead, the most commonly used rapid diagnostic method for detecting *T. marneffei* infections in urine samples is immunological testing. **Table 4** presents studies using urine for the diagnosis of talaromycosis.

**Table 4:** Sensitivity and specificity of different detection techniques in urine samples.

Sample Type	Patients	Techniques	Targets	Sensitivity (%)	Specificity (%)	Time	Ref
Urine	37 culture-proven TM patients (HIV+)	Dot blot ELISA	*T. marneffei* antigen	94.59 (35/37)	97.33 (292/300)	N/A	(Desakorn et al., 2002)
		ELISA	*T. marneffei* antigen	97.30 (36/37)	98.00 (294/300)		
		Latex agglutination test	*T. marneffei* antigen	100.00 (37/37)	99.33 (298/300)		
	33 culture-proven TM patients (HIV+)	ELISA	*T. marneffei* antigen	96.97 (32/33)	98.00 (294/300)	N/A	(Desakorn et al., 1999)
	74 culture-proven TM patients	MAb 4D1-GNA sandwich ELISA	Cytoplasmic yeast antigen	89.19 (66/74)	98.69 (226/229)	17 hours	(Shu et al., 2023)
	66 culture-proven TM patients (HIV+)	Immunochromatographic test (MAb 4D1)	Cytoplasmic yeast antigen	87.87 (58/66)	100.00 (112/112)	20 min	(Pruksaphon et al., 2018)
	76 culture-proven TM patients	immunochromatographic strip test (GNA-MAb 4D1 sandwich ELISA)	Cytoplasmic yeast antigen	89.47 (68/76)	100.00 (265/265)	30 min	(Pruksaphon et al., 2021)

A dot blot ELISA and a latex agglutination (LA) test using the same polyclonal antibody were developed to detect *T. marneffei* in urine. The LA test demonstrated the highest sensitivity (100%) and specificity (99.3%), showing that this simple, robust, and rapid method could enhance the diagnosis of talaromycosis (Desakorn et al., 2002). An ICT using the monoclonal antibody 4D1 conjugated with gold colloid for detecting *T. marneffei* yeast antigens in urine has also been developed. The ICT produced results within 20 minutes, and demonstrated high sensitivity, specificity, and accuracy of 87.87%, 100%, and 95.5% respectively (Pruksaphon et al., 2018). Another study developed a sandwich ELISA technique using monoclonal antibody 4D1 and *Galanthus nivalis* agglutinin (GNA) to detect *T. marneffei* cytoplasmic yeast antigen in human urine, achieving a sensitivity of 89.19% and a specificity of 98.69% (Shu et al., 2023). In a previous study, the MAb-Mp1p ELISA assay proved to have a superior sensitivity compared to blood culture, and the performance of the MAb-Mp1p assay exhibited higher sensitivity with urine samples compared to plasma samples (Thu et al., 2021). These studies demonstrate that immunologic tests can rapidly, easily and effectively diagnose talaromycosis using urine.

### 4.4 Cerebrospinal fluid

Cerebrospinal fluid (CSF) has unique characteristics that make it a critical specimen for detecting *T. marneffei* in the central nervous system (CNS) involvement. Given the low volume of CSF generally available for testing and the often-lower concentration of pathogens present, molecular methods such as PCR-based tests are preferred for their high sensitivity and specificity (Yamamoto, 2002). However, the clinical manifestations of *T. marneffei* in the CNS are nonspecific and CNS infections are relatively rare. As a result, such infections are often easily overlooked in clinical setting. While there are no standardized diagnostic protocols for talaromycosis in CSF, molecular methods have been applied and published as case reports. For example, a 65-year-old man initially misdiagnosed with herpetic encephalitis was later found to have a co-infection with *T. marneffei* and *Aspergillus niger* using mNGS and PCR, enabling a change to appropriate treatment (Gao et al., 2022). In another case, an immunocompetent patient with an unclear CNS condition was diagnosed with *T. marneffei* following mNGS testing of a CSF sample (Tsang et al., 2021). These cases highlight the potential for mNGS in pathogen detection, particularly when conventional testing methods are insufficient. To date, there is limited information with immunological tests for *T. marneffei* using CSF. However, Li and colleagues reported a patient in which a diagnosis of talaromycosis was achieved by targeting the Mp1p antigen using an immunofluorescence method (Li et al., 2024). Overall, the diagnosis of talaromycosis by molecular or immunological approaches remains in its infancy.

### 4.5 Skin and other infected tissues

As described in Section 1.4, skin manifestations are common in *Talaromyces* infections, which can be readily diagnosed based on histopathology and culture. For patients suspected of talaromycosis, dermatoscopy can provide an initial presumptive diagnosis. Dermoscopy magnifies and illuminates the skin for enhanced observation. For example, a 24-year-old male patient with nonspecific skin lesions was examined using dermatoscopy, which showed “circular or quasi-circular whitish amorphous structure with a central brownish keratin plug” inconsistent with the initial presumptive diagnosis of a syringoma. This finding led to a biopsy and scrapings, which subsequently grew *T. marneffei* (Xian et al., 2019).

In addition to the skin, other tissues, like lymph nodes, bone marrow, osteolysis, lung tissue, and glandular tissues can be tested to determine the presence of *T. marneffei* infection (Pruksaphon et al., 2020b). For example, 14 patients with osteolytic lesions were diagnosed with disseminated *T. marneffei* infection by histological analyses of bone biopsies using PAS or WGS (Qiu et al., 2015). The combination of the WGS and PAS staining has been suggested to enhance the accuracy of identification of *T. marneffei* in cases of bone marrow smear samples to better differentiate *T. marneffei* from other intracellular fungi (Qin et al., 2015). However, this approach is not sufficient to distinguish *T. marneffei* from *Histoplasma capsulatum*, and additional cultures or other diagnostic tests are required for the definitive diagnosis of these pathogens (Chang et al., 1995). Moreover, accurate microscopic analysis demands skilled laboratory professionals and often requires verification by at least two independent analysts to confirm the results.

MAbs designed for use with blood samples often encounter limitations when applied to tissue samples due to differences in antigen presentation and the impact of tissue processing, such as antigen masking by fixatives like formalin. Additionally, the complex tissue environment, including diverse cell types and interfering proteins, can reduce the effectiveness and specificity of these antibodies. Consequently, immunological testing methods are generally less effective in diagnosing *T. marneffei* in tissue samples compared to blood samples. However, a study has demonstrated the efficacy of indirect immunofluorescence assays using polyclonal rabbit antibodies for identifying *T. marneffei* in cultures and tissue specimens, showing specificity to the yeast form of the fungus after appropriate absorption (Kaufman et al., 1995). Building upon these findings, ongoing research has focused on the development and characterization of MAbs with potential diagnostic utility for identifying *T. marneffei* in tissue samples (Trewatcharegon et al., 2000). However, the advancement and application of immunological methods in tissue samples have not progressed as rapidly as those for blood and urine samples.

Molecular detection methods have been applied to the diagnosis of talaromycosis, but they are not standardized for routine use. For example, PCR was first used in 2003 to direct *T. marneffei* from a skin biopsy specimen (Tsunemi et al., 2003). In another study, paraffin-embedded tissue samples, including 14 cases from various anatomical sites such as skin, lungs, bronchi, and lymph nodes, were subjected to a Nested PCR. The detectable DNA concentrations for single PCR or nested PCR were 14 pg/μL and 14 fg/μL, respectively (Zeng et al., 2009). In a BALB/c nude mice model, a nested PCR demonstrated both sensitivity and specific for detecting *T. marneffei* in BALF and fresh tissues (Liu et al., 2016). In another study, a LAMP technique was developed and evaluated for the rapid diagnosis of *T. marneffei* in archived tissue samples, which including skin, lung, and lymph nodes, and the approach was shown to be specific for *T. marneffei* and excluded related pathogens (Sun et al., 2010). mNGS has been applied to diagnosing *T. marneffei* in biopsies, but the approach has not been standardized. For example, mNGS successfully identified *T. marneffei* in lymph node biopsy tissues from patients with immune deficiencies (Liu et al., 2022b). mNGS can be useful in establishing a diagnosis of fever of unknown origin, such as with a 22-year-old male who presented with non-specific symptoms including fever, cough, weakness, jaundice, and rash, where extensive conventional diagnostic methods failed to determine the underlying cause. Through mNGS analysis of skin tissue, bone marrow, blood, and cerebrospinal fluid samples, multiple *T. marneffei* nucleotide sequences were detected to confirm disseminated *T. marneffei* infection (Zhu et al., 2018). In another reported case involving an HIV-infected patient presenting with fever and abdominal pain, *T. marneffei* was rapidly identified through mNGS of FFPE tissue samples from the omentum majus (Zhou et al., 2021). However, the widespread implementation of mNGS is currently limited by its high costs and the socioeconomic constraints prevalent in regions that would benefit most from advanced diagnostic tools. Therefore, future research should focus on strategies to reduce diagnostic costs and expand DNA libraries for mNGS, which could significantly improve the accessibility and clinical utility of this technology.

## 5 Combining diagnostic methods

The diagnosis of *T. marneffei* using a single non-culture-based technique is a matter that requires deep and careful consideration, as its diagnostic performance is often limited particularly in immunocompromised patients, where early and accurate diagnosis is crucial. To mitigate these shortcomings, combining non-culture-based methods has been established as a strategy to enhance diagnostic performance. One effective approach is the combination of antigen and antibody detection. For instance, assays targeting the Mp1p antigen have shown promising results. While Mp1p antigen detection alone offers good sensitivity, not all patients may have detectable levels of circulating antigen at the time of testing. By concurrently testing for anti-Mp1p antibodies, the diagnostic yield is significantly improved, as some individuals may not have detectable antigen levels but may have already developed a measurable antibody response. A study conducted by Wang et al. indicated that Mp1p antigen and antibody tests complement one another, Some individual serum samples showed significant levels of both Mp1p antigen and anti-Mp1p antibodies simultaneously. This complementary relationship is crucial, as combining both test results increased the diagnostic sensitivity to 100% in patients with talaromycosis (Wang et al., 2011). Another potent combination involves antigen detection paired with molecular techniques like quantitative PCR (qPCR). Antigen detection assays, particularly those based on lateral flow procedure, provide rapid results and are suitable for initial screening. However, their specificity may be reduced by antibody cross-reactivity with other pathogenic fungi. To enhance diagnostic precision, qPCR assays targeting *T. marneffei*-specific genetic regions—such as the ITS region, MP1 gene cluster or 5.8S rRNA—can be employed to verify the presence of fungal DNA with high specificity (Woo et al., 2007; Khanh et al., 2025). This synergy facilitates both rapid screening and definitive molecular confirmation.

## 6 Conclusion

Traditionally, the *T. marneffei* diagnostic gold standard has relied heavily on culturing techniques, but these methods are time-consuming and less than ideal given the rapid progression of the infection in vulnerable patients. Non-culture-based diagnostic techniques have led to the development of various methods that provide faster detection of *T. marneffei*. These methods have improved turnaround times and been adapted to allow high-throughput testing. Despite significant advancements in diagnostic technologies, the selection of an appropriate diagnostic method for *T. marneffei* often relies on available resources, the clinical setting, and specific characteristics of the patient’s conditions (Castro-Lainez et al., 2018; Ning et al., 2018). Crucially, there is no universal consensus on the optimal rapid diagnostic technique due to variations in sensitivity and specificity among different methods, particularly when different types of clinical specimens are considered. Each specimen type—ranging from blood, and urine to more invasive samples like CSF and tissue biopsies—may require distinct processing and diagnostic approaches to accurately detect *T. marneffei*. Furthermore, challenges related to sensitivity and specificity remain and significantly vary depending on methodology and sample type. Additionally, the high cost of non-culture-based technologies limits their widespread application in China, Thailand, and Vietnam, where talaromycosis is most prevalent. Continued research and development are needed to improve these diagnostic tools and increase their accessibility, thereby improving patient outcomes. Future research should also focus on integrating these diagnostic methods into routine clinical practice to streamline the testing process and reduce reliance on culture methods, which are time-consuming and often less sensitive. Furthermore, advancements in non-culture diagnostic methods for *T. marneffei* infections are crucial for enhancing diagnostic efficiency and accuracy, especially in resource-limited settings. By reducing the time and expertise needed compared to traditional culture methods, these techniques enable faster identification and treatment, which is crucial for immunocompromised populations. Importantly, combining multiple diagnostic techniques can help overcome the limitations of individual methods, leading to more comprehensive and rapid detection. However, efforts to further improve the most effective technique are essential. These innovations align with the United Nations Sustainable Development Goals (UN-SDGs), particularly in improving healthcare accessibility, diagnosis, and treatment outcomes, as well as promoting the development of rapid, scalable diagnostic technologies (FAO et al., 2023).

Ultimately, we would like to emphasize that the selection of non-culture-based diagnostic methods for *T. marneffei* must be guided by rational decision-making and a multidimensional assessment. This is particularly important in the current era of global uncertainty, marked by trade conflicts, economic competition, reciprocal tariffs, IP access restrictions and shifting geopolitical dynamics. Accordingly, we have compiled a summary table (**Supplementary Table 1**) that categorizes the available non-culture-based diagnostic approaches based on key consideration factors, including cost, reliability, convenience, and accessibility, in relation to the epidemiological context of *T. marneffei* infection.
