# Supplementary material for: An overview of rapid non-culture-based techniques in various clinical specimens for the laboratory diagnosis of Talaromyces marneffei
Source: Front Cell Infect Microbiol. 2025 May 23;15:1591429. doi: 10.3389/fcimb.2025.1591429 (PMC12141349; doi:10.3389/fcimb.2025.1591429)
Supplement: Supplementary file 1 [file Table1.docx]

**Supplementary Table 1. Recommendation table for non-culture-based diagnostic methods for *T. marneffei***

| A. Endemic and Underdeveloped Regions (e.g., rural Southeast Asia, some areas in mainland China and India)  Priorities: Low cost, simplicity, minimal infrastructure | |
| --- | --- |
| Recommended Diagnostic Methods | Consideration factors |
| Microscopic based + Histopathology (stained tissue, skin scrapings, bone marrow) | Rapid, low-cost, but less sensitive; widely available in basic laboratories. |
| Lateral Flow Immunochromatographic Assays (LFA) for *T. marneffei* Antigen | Rapid, affordable, no need for equipment; suitable for bedside or low-resource settings. |
| In-house ELISA for Antigen Detection | Low cost if developed locally; moderate sensitivity and specificity; depends on reagent availability. |
| B. Endemic and Developed Regions (e.g., urban Thailand, Vietnam, Southern China with tertiary hospitals) Priorities: Balance between diagnostic performance and cost, better infrastructure | |
| Recommended Diagnostic Methods | Consideration factors |
| Antigen Detection ELISA or LFA | High sensitivity/specificity, rapid turnaround. |
| Real-Time PCR (qPCR) | Highly sensitive and specific; used in central labs or reference hospitals. |
| Multiplex PCR (for differential fungal diagnosis) | Efficient in co-endemic areas with overlapping symptoms (e.g. Fever of unknown origin). |
| Next-Generation Sequencing (NGS) | Optional for complicated or atypical cases; not routine due to cost and technical expert. |
| C. Non-Endemic and Developed Regions (e.g., US, Europe, Japan) Priorities: High diagnostic accuracy, used in imported/mycosis cases | |
| Recommended Diagnostic Methods | Consideration factors |
| qPCR with Specific Primers (e.g., ITS region or MP1 gene of *T. marneffei*) | \| Preferred method for rare/atypical cases; very sensitive and specific. \| \| --- \| |
| NGS/Metagenomic Sequencing | Used when fungal infection is suspected but not identified; effective in BALF, blood, tissue. |
| Serology (IgM/IgG, especially in travelers) and GM or BDG assay | Helpful in retrospective diagnosis or screening.  GM and BDG are non-specific but widely used as fungal markers, especially in screening. |
| Antigen Detection Assays (if accessible via reference labs) | Turnaround may be delayed, but useful. |
| Histopathology with specialized staining (e.g. Gomori Methenamine Silver and Periodic acid-Schiff) | It is still important when fungal forms are visualized in tissue biopsies. |

**Supplementary Table 1. Recommendation table for non-culture-based diagnostic methods for *T. marneffei* (Continue)**

| **D. Non-Endemic and Underdeveloped Regions** (e.g., refugee camps, migrant health settings in non-endemic areas)  **Priorities:** Simplicity, portability, rapid testing | |
| --- | --- |
| **Recommended Diagnostic Methods** | **Consideration factors** |
| Lateral Flow Assay (if available) | Ideal due to low resource demand. |
| Microscopy and Histopathology (skin, sputum, bone marrow smears) | \|  \| Most accessible but low sensitivity and specificity. \| \| --- \| --- \| |
| Referral to regional labs for PCR or ELISA | In cases with high suspicion and other clinical symptoms compatible with talaromycosis. |
| **Note:** ELISA, PCR based and other sophisticated assays are often not available here due to lack of infrastructure and trained laboratory technicians. | |
